# So close, yet so far away: the relationship between MAM and cardiac disease

**DOI:** 10.3389/fcvm.2024.1353533

**Published:** 2024-02-05

**Authors:** Bo Lu, Xiaozhe Chen, Yulong Ma, Mingtai Gui, Lei Yao, Jianhua Li, Mingzhu Wang, Xunjie Zhou, Deyu Fu

**Affiliations:** ^1^ Department of Cardiology, Yueyang Hospital of Integrated Traditional Chinese and Western Medicine, Shanghai University of Traditional Chinese Medicine, Shanghai, China; ^2^ Division of Nephrology and Hypertension, Mayo Clinic, Rochester, MN, United States; ^3^ Shanghai University of Traditional Chinese Medicine, Shanghai, China

**Keywords:** mitochondria-associated membrane, cardiac disease, Ca^2+^ homeostasis, apoptosis, autophagy

## Abstract

Mitochondria-associated membrane (MAM) serve as crucial contact sites between mitochondria and the endoplasmic reticulum (ER). Recent research has highlighted the significance of MAM, which serve as a platform for various protein molecules, in processes such as calcium signaling, ATP production, mitochondrial structure and function, and autophagy. Cardiac diseases caused by any reason can lead to changes in myocardial structure and function, significantly impacting human health. Notably, MAM exhibits various regulatory effects to maintain cellular balance in several cardiac diseases conditions, such as obesity, diabetes mellitus, and cardiotoxicity. MAM proteins independently or interact with their counterparts, forming essential tethers between the ER and mitochondria in cardiomyocytes. This review provides an overview of key MAM regulators, detailing their structure and functions. Additionally, it explores the connection between MAM and various cardiac injuries, suggesting that precise genetic, pharmacological, and physical regulation of MAM may be a promising strategy for preventing and treating heart failure.

## 1 Introduction

The interplay among organelles is the fundamental framework for cellular signaling. Approximately 5%–20% of the outer mitochondrial membrane (OMM) intimately interfaces with the endoplasmic reticulum (ER), maintaining a distance within the range of 10–30 nm and forming a specialized structure termed the mitochondrial-associated endoplasmic reticulum membrane (MAM) (1). Proteomic analysis has unveiled the presence of approximately 1,000–2,000 distinct proteins within the MAM, highlighting its role as a multifaceted signaling platform (2, 3). The MAM orchestrates bidirectional regulation of organelle functions, thereby exerting influence over a spectrum of cellular processes, including energy metabolism, calcium (Ca^2+^) handling, lipid balance, and the intricate control of cell survival and apoptosis. Furthermore, it is a central hub (4) for signaling in regulating mitochondrial fission. Cardiac diseases due to any cause, such as diabetes, obesity, hypertension, and myocardial ischemia‒reperfusion (I/R) injury, can increase the risk of heart failure and cardiovascular mortality. Recent investigations have underscored the pivotal role played by MAM in these processes, and this article provides an overview of the latest developments in this field.

## 2 The main biological functions of MAM

MAM, a highly dynamic structure, functions as a cellular bridge, coordinating the exchange of substances and signaling molecules. It plays a pivotal role in preserving the physiological functions and metabolic balance of cells. Complex regulatory mechanisms intricately govern these structures and functions under physiological conditions and stress responses.

### 2.1 MAM mediates ER-mitochondria communication and Ca^2+^ homeostasis

Diverse proteins and associated complexes within the MAM play pivotal roles in maintaining the appropriate flow of Ca^2+^ and essential physiological functions between the ER and mitochondria. Precise Ca^2+^ transfer not only governs mitochondrial bioenergetics but also regulates processes such as mitochondria-mediated cell death, autophagy/mitophagy, dynamics of mitochondrial fusion and fission, generation of reactive oxygen species (ROS), and transmission of redox signals (5). In mammals, the ER-resident chaperone glucose-regulated protein 75 (GRP75) forms a complex with the ER transmembrane Ca^2+^ release channel, inositol 1,4,5-trisphosphate receptor (IP3R), and the OMM protein voltage-dependent anion channel (VDAC), establishing an efficient transfer of Ca^2+^ from the ER to mitochondria (6). Various stimuli can dynamically influence the stability of this complex, favoring the uptake of Ca^2+^ into mitochondria through direct release via the IP3R channel (6). Once Ca^2+^ traverses the OMM, it enters the mitochondrial matrix via the mitochondrial Ca^2+^ uniporter (MCU) complex (including MCU, MCUb and regulatory subunits), mitochondrial ryanodine receptor (RyR) or rapid mode Ca^2+^ uptake (7, 8). However, when external stimuli enhance the interplay between the ER and mitochondria through IP3R-GRP75-VDAC1 complex (6, 9), a substantial amount of Ca^2+^ is absorbed by mitochondria, stimulating the opening of the mitochondrial permeability transition pore (mPTP), which leads to the release of cytochrome C and a cascade of apoptotic events (10). Phosphofurin acidic cluster sorting protein 2 (PACS-2) within MAM facilitates Ca^2+^ transfer between the ER and mitochondria and plays a role in regulating lipid synthases and autophagy (11). The depletion of PACS-2 increases the separation between these two organelles and triggers apoptosis by promoting the cleavage of B-cell receptor-associated protein 31 (BAP31) (12). BAP31 promotes apoptosis by mobilizing ER calcium stores at MAM and regulates mitochondrial function via interaction with mitochondrial fission protein 1 (Fis1) (13) or mitochondrial membrane 40 (Tom40) (14), respectively. Mitochondrial fusion proteins (MFNs), including MFN1 and MFN2 (15), are involved in the regulation of mitochondrial Ca^2+^ (_m_Ca^2+^). MFN1 is predominantly located in the OMM, while MFN2 is found in both the OMM and ER membranes (16, 17). MFN2 forms heterotypic or homotypic complexes with MFN1 or MFN2, facilitating the bridging of the ER and mitochondria (16). Additionally, protein complexes such as RyR 2 and VDAC2 are also present in cardiac MAM and play a role in regulating Ca^2+^ transfer (18).

### 2.2 MAM regulates mitochondrial metabolism

Mitochondria, often called powerhouses, occupy approximately 40% of the volume in adult cardiomyocytes and generate approximately 90% of the cell's adenosine triphosphate (ATP) (19). MAM significantly influences mitochondrial respiratory function, thereby contributing to maintaining mitochondrial metabolic homeostasis (20). The concentration of _m_Ca^2+^ serves as a central signaling mechanism that regulates mitochondrial metabolism (21, 22) by governing the activities of key enzymes such as pyruvate dehydrogenase/phosphatase, isocitrate dehydrogenase, alpha-ketoglutarate dehydrogenase, and ATP synthase. These enzymes promote the production of reduced nicotinamide adenine dinucleotide (NADH) and reduced flavin adenine dinucleotide, which, in turn, sustain the functionality of the electron transport chain (23). In addition, ER stress can induce changes in the conformation of the ER, thereby altering the distribution of mitochondria around the ER and increasing the contact points between these two organelles. Adaptive modifications of the MAM structure facilitate Ca^2+^ exchange and ATP production in response to stressors (24). However, loss of Ca^2+^ transfer results in impaired mitochondrial metabolism, leading to increased phosphorylation of enzymes, rendering them inactive, which also slows down the tricarboxylic acid cycle, ultimately compromising the production of mitochondrial ATP (25). When cellular ATP levels become insufficient to support basic metabolic processes, cell necrosis can occur (26). At rest, the relatively low activity of the MCU in the inner mitochondrial membrane (IMM) is adequate to maintain baseline ATP levels. However, when stressors trigger rapid heartbeats, there is an increase in MCU-dependent _m_Ca^2+^ uptake (27), accompanied by intensified NADH fluorescence signals (28). These changes indicate an elevation in mitochondrial metabolism (29), followed by a significant increase in ATP synthesis after an initial 10% sharp decline. This indicates that the regulation of cellular energy homeostasis by MAM involves intricate and finely tuned mechanisms.

## 3 Role of MAM in cardiac disease

### 3.1 Diabetic cardiomyopathy

**Figure 1:**
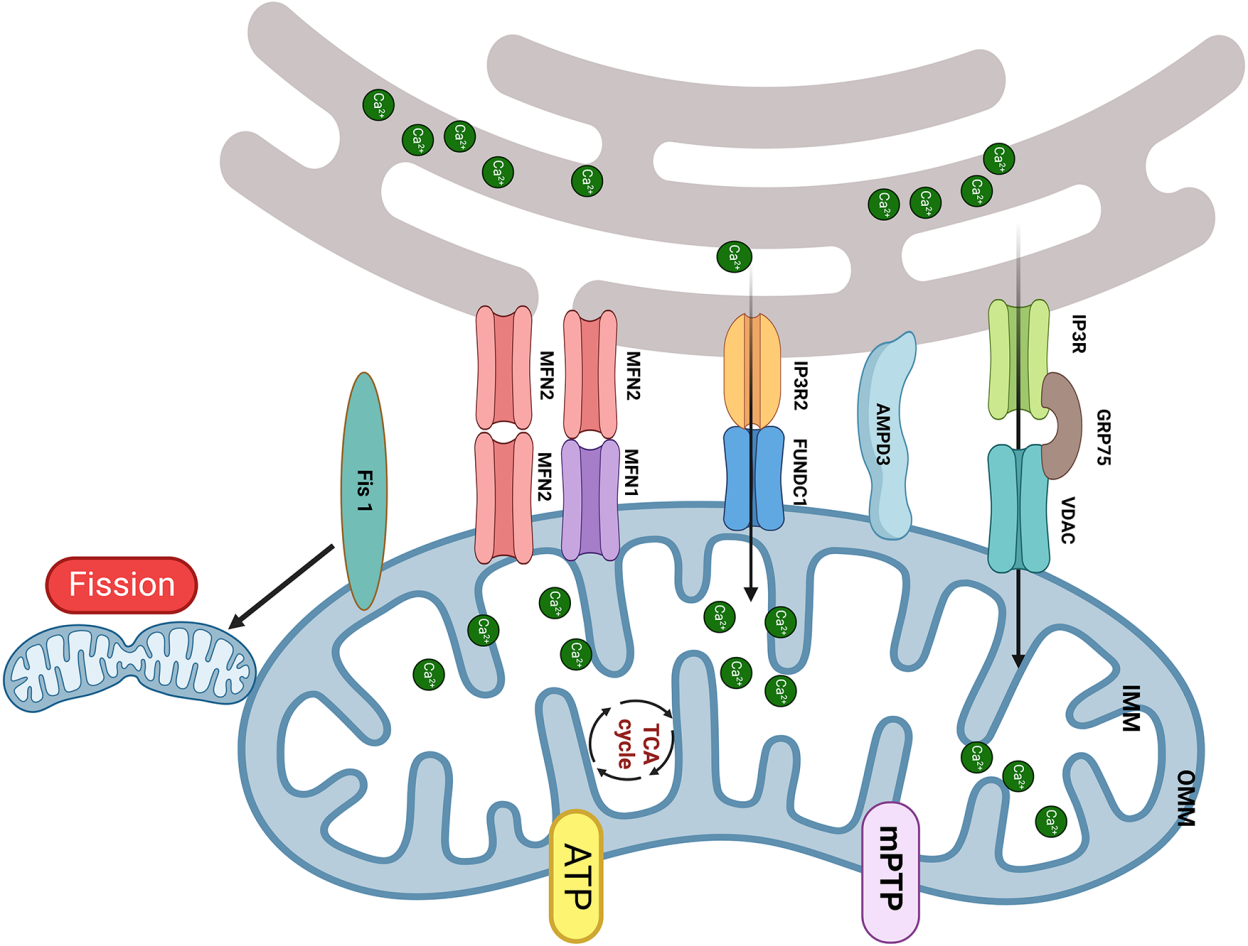
The MAM proteins involved in DbCM. (1) The upregulation of FUNDC1 and IP3R2 leads to _m_Ca^2+^ overload results in mitochondrial fragmentation, mPTP opening, and cardiomyocyte apoptosis; (2) Increased expression of Fis1 in DbCM leads to excessive mitochondrial fission, causing myocardial injury; (3) Disruption of the interaction between IP3R and VDAC is accompanied by a decrease in _m_Ca^2+^ and mitochondrial bioenergetics, results in myocardial injury; (4) The upregulation of MFN2 and AMPD shorten the distance of MAM and induced mitochondrial dysfunction. Created with BioRender.com.

By 2050, it is estimated that more than 1.31 billion individuals will suffer from diabetes (30). This condition has sparked significant interest because of its serious complications, especially diabetic cardiomyopathy (DbCM). Numerous studies have highlighted that change in the structure of MAM and the consequent dysfunction of mitochondria are pivotal mechanisms of DbCM. This mechanism is complex and involves various cell types and proteins, and some research results are still under dispute (Figure 1).

Compared to nondiabetic individuals, diabetic patients exhibit a significant elevation in FUN14 domain containing 1 (FUNDC1) expression within their cardiac tissues (31). In vitro, high glucose (HG) can increase FUNDC1, IP3R2, and MAM levels in neonatal mouse cardiomyocytes, resulting in increased _m_Ca^2+^ (31, 32). Elevated _m_Ca^2+^ concentration adversely leads to mitochondrial fragmentation, prolongation of mPTP opening duration, and the initiation of cell apoptosis. The transgenic downregulation of FUNDC1 or IP3R2 effectively hindered the formation of MAM, decreased Ca^2+^ flux, and ameliorated mitochondrial dysfunction in cardiomyocytes exposed to HG. Additionally, the interaction between FUNDC1 and IP3R2 inhibits the ubiquitination and protease-mediated degradation of IP3R2. In vivo, cardiomyocyte-specific deficiency of FUNDC1 in diabetic mice abolishes MAM formation, mitigates the increase in _m_Ca^2+^, and enhances both mitochondrial and cardiac function. Moreover, diabetes prompts the expression of Fis1 within cardiac mitochondria, leading to excessive mitochondrial fission and consequent disruption of cardiac structure and function. Knocking out FUNDC1 can maintain normal mitochondrial morphology and enhance cardiac structure and function by suppressing cardiomyocyte Fis1 expression. Consequently, the absence of FUNDC1 in cardiomyocytes ameliorates cardiac dysfunction by lowering _m_Ca^2+^ levels and mitigating mitochondrial fission. Furthermore, FUNDC1 can interact with light chain 3 (LC3), serving as a mitophagy receptor. However, it is worth noting that mitophagy is not upregulated in DbCM, which suggests that FUNDC1 mediates DbCM through a pathway independent of mitophagy.

In contrast, diabetic mice fed a 16-week high-fat high-sugar diet (HFHSD) had a notable decrease in the interaction between IP3R and VDAC within the cardiac tissue (33). This alteration is accompanied by a decline in Ca^2+^ transfer and mitochondrial bioenergetics, resulting in reduced cardiomyocyte contractility (33). The expression of ER-mitochondrial junctions via adenovirus-mediated delivery could rescue this situation. Notably, the levels of ER Ca^2+^, cytosolic Ca^2+^ transients, and MCU function remained unchanged. In vitro, a reduction in the IP3R/GRP75/VDAC Ca^2+^ channel complex was reduced in HFHSD cardiomyocytes, along with a decrease in IP3R-stimulated Ca^2+^ transfer to mitochondria (33). An 8-week diet reversal can restore normal cardiac function and prevent the progression of DbCM by mitigating MAM-mediated _m_Ca^2+^ dysfunction (33). Therefore, the diminished transfer of Ca^2+^ from the ER to mitochondria appears to be a reversible trigger for mitochondrial dysfunction in DbCM. Of note, Fauconnier et al. (34) reported that both HFHSD and ob/ob diabetic mice had a reduction in IP3-driven _m_Ca^2+^ transport, which suggested that the duration of diabetes may exert a more significant influence on MAM function than the causes, emphasizing the need for further research into its potential mechanisms.

MFN2 also plays a pivotal role in the process of DbCM. In vitro, HG can accelerate ER stress (ERS) and mitochondrial oxidative stress and lead to the accumulation of MAM proteins, especially GRP75 and MFN2, within primary cardiomyocytes (35). The ERS inhibitor sodium 4-phenylbutyrate can counteract these changes. The distance of MAM is reduced when HL-1 cells are exposed to ERS, which is reversed by MFN2 siRNA (35). The disruption of MAM plays a crucial role in preventing mitochondrial dysfunction due to _m_Ca^2+^ overload and then protects cells from ERS-induced apoptosis (35). Furthermore, HG also induces the formation of MAM in H9c2 cells, which results in a reduction in mitochondrial biogenesis, fusion, and oxidative phosphorylation. The downregulation of critical components in mitochondrial respiratory complexes resulted in ATP deficiency, leading to subsequent release of apoptotic proteins from mitochondria, along with a shortage of antiapoptotic proteins (32). These findings underscore that MAM-mediated mitochondrial impairments can induce apoptosis in cardiomyocytes.

Adenosine monophosphate deaminase (AMPD3) impairs diastolic function in pressure-overloaded type 2 diabetic hearts by reducing ATP production (36). The same group also found that the 90-kDa AMPD3 protein is present in the OMM/ER and MAM. In addition, its levels are significantly higher in the cardiac tissue of obese diabetic model rats (Otsuka Long-Evans Tokushima Fatty, OLETF)) than in control rats (Long-Evans Tokushima Otsuka, LETO). Following dobutamine-induced pressure overload, OLETF rats display diastolic dysfunction, a 57% increase in MAM area and a 47% elevation in _m_Ca^2+^ level compared to LETO rats. Intriguingly, before pressure overload, the Ca^2+^ retention capacity in MAM-containing crude mitochondria of OLETF was 21% lower than that of LETO. Moreover, *in vitro* transfection of FLAG-AMPD3 in cells promotes the formation of MAM, resulting in _m_Ca^2+^ overload, mitochondrial dysfunction and impaired mitochondrial respiration (37).

The function of T cells is also linked with myocardial fibrosis (38, 39). The ketogenic diet (KD) reduced regulatory T cells (Tregs) proportion in plasma and induced cardiac diastolic disfunction and fibrosis in db/db mice (40). Moreover, culture medium from ketone bodies (KBs)-treated Tregs can synergistically activate cardiac fibroblasts. KBs inhibited the differentiation and proliferation of naive CD4+ T cells into Tregs, and hampered the MAM, mitochondrial respiration, fatty acid oxidation within Tregs. Improving T-cell function will simultaneously enhance MAM and mitochondrial respiratory function (40). Therefore, MAM dysfunction of Tregs might play a indirectly role in KD induced diabetic myocardial fibrosis, and its direct effect on Tregs needs to futher clarified.

### 3.2 Obesity-related cardiomyopathy

As obesity rates continue to rise, attention is increasingly focused on the associated myocardial injuries. Mice with metabolic syndrome (MS) induced by prolonged consumption of a Western diet often exhibit cardiac microvascular dysfunction, mitochondrial damage, and cardiac remodeling (41). The reshaping and deformation of mitochondria and the ER markedly reduce MAM size in MS mouse cardiomyocytes. Additionally, it has been noted that the expression of MFN2 in the hearts of MS mice is significantly decreased compared to the control group. Altogether, these data suggest that obesity can disrupt communication between the ER and mitochondria (41). Furthermore, it has been reported that the deletion of FUNDC1 aggravates cardiac remodeling, mitochondrial dysfunction, and cell death in high-fat diet (HFD)-fed mice. The absence of FUNDC1 also reduces ER Ca^2+^-regulating protein IP3R3 degradation by disrupting its interaction with F-Box and leucine rich repeat protein 2 (FBXL2), which exacerbates cardiac structural and functional abnormalities and mitochondrial dysfunction through Ca^2+^ overload (42). This study also reveals that both autophagy and mitophagy levels are suppressed in the hearts of obese mice. Systemic FUNDC1 knockout enhances the loss of autophagy induced by HFD but has little impact on other mitophagy proteins, which suggests that FUNDC1-mediated mitophagy uniquely affects HFD-induced cardiac abnormalities (42). Thus, FUNDC1 is a unique therapeutic target in obesity-induced cardiac abnormalities indirectly through the FBXL2-IP3R3 axis. In addition, syntaxin 17 (STX17), a scaffold protein located in MAM, acts as a central hub for physical and functional interactions between the ER and mitochondria and is also involved in autophagy (43) and mitophagy (44). The levels of STX17 are significantly elevated in the plasma of obese patients and in the heart tissues of mice fed with HFD. This elevation promotes _m_Ca^2+^ overload, leading to obesity-related cardiomyopathy in a Parkin-dependent manner. Deletion and overexpression of STX17 mitigate and exacerbate HFD-induced cardiac oxidative stress, mitochondrial damage, and functional impairments, respectively (45). In summary, MAM may be involved in obesity-related cardiomyopathy either independently or through autophagy.

### 3.3 Myocardial ischemia–reperfusion injury

_m_Ca^2+^ overload plays a pivotal role in myocardial I/R injury (46), giving rise to disruptions of oxidative phosphorylation, the generation of ROS, and the opening of the mPTP (47). mPTP is one of the crucial mediators to cell death in I/R (48), whereas the exact molecular composition of mPTP is still controversial (49), e.g., the double knock-out of adenine necleotide translocator (ANT)1/2 or triple ANT1, 2, 4 genes have different effects on mPTP activity (49, 50). Cyclophilin D (CYPD) within the mitochondrial matrix has been identified as a pivotal regulator of the pore opening (51), and its interaction with IP3R1 in cardiomyocytes is intensified under conditions of hypoxia-reoxygenation (H/R) (52). Notably, CYPD deficiency can confer protection to the heart against I/R injury and necrosis (53). The knockout of CYPD can induce anomalies in IP3R-mediated Ca^2+^ transport from the ER to the mitochondria (54). Furthermore, genetic deletion of the PPIF gene, which is responsible for encoding CYPD in cardiomyocytes, disrupts the interaction between CYPD and the VDAC1/GRP75/IP3R1 complex, mitigating the _m_Ca^2+^ load induced by H/R (52). Similarly, pharmacological or genetic inhibition of CYPD, IP3R1, or GRP75 can shield cardiomyocytes from cell death and _m_Ca^2+^ overload caused by H/R (52).

In the hearts of mice, a close association is observed between NADPH Oxidase 4 (NOX4) and MAM-labeled FACL4 in the ER region proximate to mitochondria, implying that NOX4 may be localized to MAM (55). During I/R, Nox4 deficiency in cardiomyocytes increases cardiac troponin I release sevenfold, exacerbating myocardial damage. NOX4 can enhance Akt-dependent phosphorylation of IP3R1, consequently inhibiting Ca^2+^ flux and mPTP-dependent necrosis. Ultimately, NOX4 reduces the extent of myocardial infarction, underscoring the pro-survival effects of NOX4, which rely on spatially confined signal transduction within MAM.

Protein tyrosine phosphatase interacting protein 51 (PTPIP51) is a phospholipid transfer protein endowed with MAM-tethering capabilities. It engages with the OMM protein PTPIP51, forming a chain of MAM with the assistance of the integral ER vesicle-associated membrane protein-associated protein B (VAPB) (56). This tether contributes to forming MAM and regulating Ca^2+^ homeostasis and autophagy (57, 58). Furthermore, PTPIP51 is significantly upregulated in hearts affected by I/R. The adenovirus-mediated upregulation of PTPIP51 substantially enhances the interaction between mitochondria and the SR, thereby augmenting _m_Ca^2+^ uptake via MCU. Conversely, MCU inhibition or genetic deletion within mitochondria can reverse the PTPIP51-induced elevation of _m_Ca^2+^ and protect cardiomyocytes from PTPIP51-induced apoptosis. Most notably, the cardiac-specific knockout of PTPIP51 significantly reduces the extent of myocardial infarction and cardiac damage following I/R injury (59). These findings suggest that PTPIP51 may represent a therapeutic target for mitigating I/R injury.

**Figure 2:**
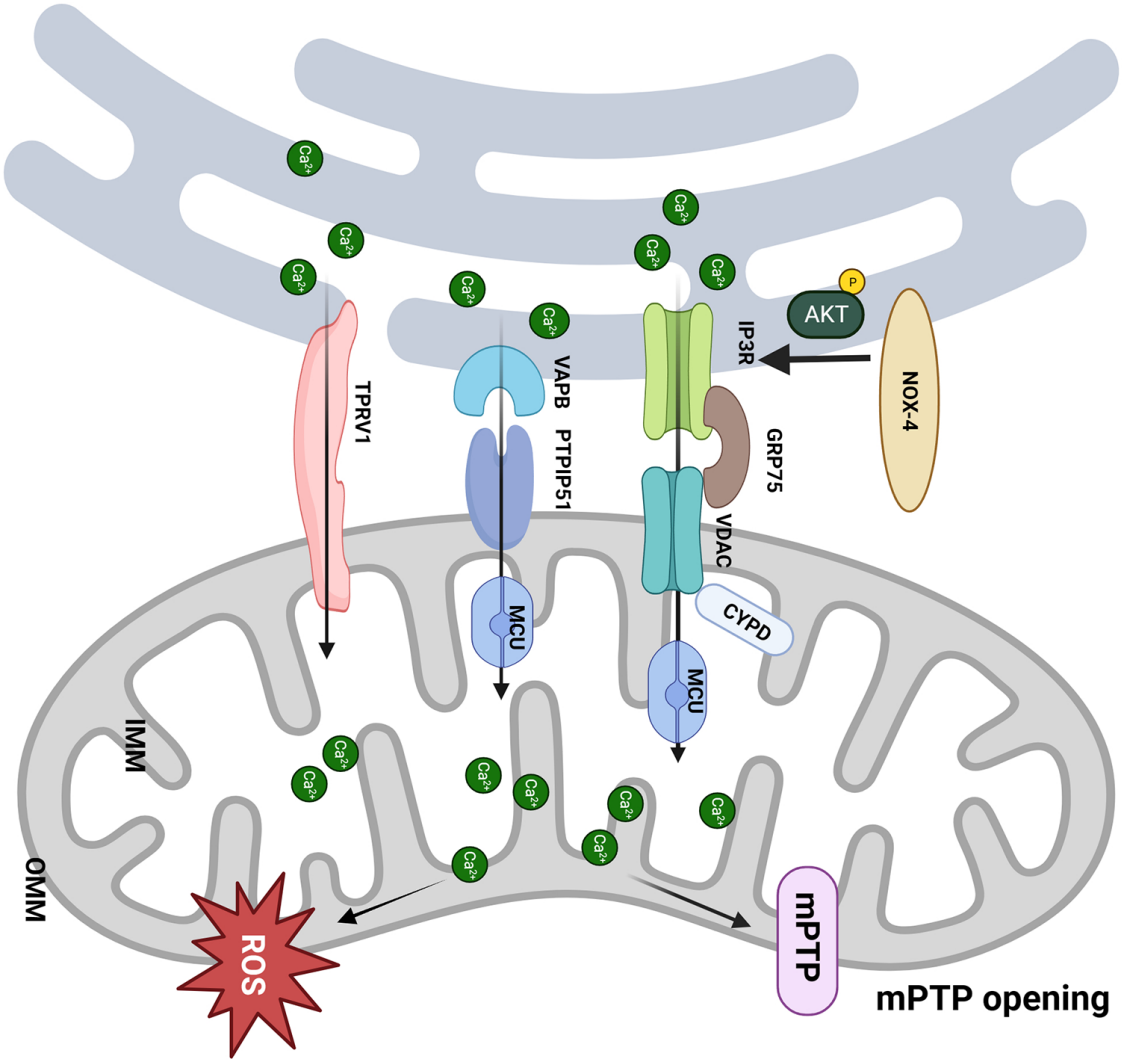
MAM participates in the pathological process of I/R. (1) The CYPD deficiency improves the _m_Ca^2+^ load and mPTP opening via interacting with the VDAC1/GRP75/IP3R1 complex, which protects the cardiomyocytes from I/R; (2) NOX4 enhances the Akt-dependent phosphorylation of IP3R1, consequently inhibits Ca^2+^ flux and mPTP-dependent necrosis; (3) Upregulation of PTPIP51 promotes interaction between mitochondria and endoplasmic reticulum and induces cell apoptosis through increasing _m_Ca^2+^ uptake by MCU; (4) Activation of TRPV1 mobilizes Ca^2+^ from ER to mitochondria and remodels MAM. Created with BioRender.com.

Nolwenn Tessier et al. demonstrated for the first time that transient receptor potential vanilloid type 1 (TRPV1) is involved in regulating MAM-mediated Ca^2+^ exchange (60). RTX, a TPRV1 agonist, slowly increased the mitochondrial Ca^2+^ content, whereas iRTX had the opposite effect. This indicates that mitochondria gradually buffered Ca^2+^ ER leakage from TRPV1 channels both inside and outside MAM. Activation of TRPV1 by RTX resulted in an elevation of Ca^2+^ concentration in specific areas on the mitochondrial surface (Ca^2+^ hot spots), which could directly mobilize Ca^2+^ from the ER to mitochondria and trigger the remodeling of MAM. Apart from Ca^2+^ transfer, sustained activation of TRPV1 also reduced the contacts between the ER and mitochondria, which might mutually decrease the Ca^2+^ content within mitochondria (60). Sun et al. demonstrated that TRPV1 activation at the beginning of H/R is harmful to cell survival, and inhibiting it protects cells from death (61). In H/R conditions, the results showed a protective effect of RTX by reducing cell mortality by ∼18% compared to control H/R only when RTX was applied before hypoxia (60). The controversial effects of the pharmacological activation of TRPV1 may be related to the treatment window, which should be finely tuned with respect to ischemia (Figure 2).

### 3.4 Hypoxia-related myocardial injury

Sleep-disordered breathing (SDB), including obstructive and central sleep apnea, causes intermittent hypoxia (IH) and reduces the survival of cardiomyocytes (62, 63). The right atrial biopsies of SDB patients and IH-exposed mice revealed that IH can activate hypoxia-inducible factor 1 (HIF-1), ER stress, and cell apoptosis (64). IH failed to elevate glucose-regulated protein 78 (GRP78) and C/EBP homologous protein (CHOP) levels in HIF-1α+/− mice. Most significantly, IH-exposed HIF-1α+/− mice exhibited a remarkable inhibition of cell apoptosis compared to HIF-1α+/+ mice. These findings strongly indicate that HIF-1 is pivotal in mediating IH-induced ER stress and cell apoptosis. In addition, IH can injure MAM, Ca^2+^ homeostasis, mitochondrial respiration, and Ca^2+^ retention capacity. Persistent MAM disruption, evidenced by reduced interaction between VDAC and IP3R1, and disturbances in Ca^2+^ homeostasis are linked to myocardial cell death (64). Notably, IH did not affect the interaction between VDAC and IP3R1 in HIF-1α+/− mice. Both *in vivo* and *in vitro* experiments found no discernible impact of IH on MAM structure and mitochondrial function in HIF-1α+/− mouse myocardium, while overexpression of HIF-1α had the opposite effect. Hence, targeting HIF-1 may hold promise for mitigating alterations in MAM and mitochondrial dysfunction induced by IH, further improving cardiomyocyte apoptosis (64).

The hypobaric hypoxic (HH) environment is a significant challenge, and prolonged exposure to HH can impair right heart function. PACS2 is crucial in regulating mitophagy. Both PACS2 and mitophagy levels are downregulated in HH mouse hearts (65). Cardiac-specific knockout of PACS2 exacerbates the inhibition of mitophagy induced by HH (65). When cardiomyocytes are exposed to HH, the absence of PACS2 disrupts MAM formation and Ca^2+^ flux between the ER and mitochondria, further inhibiting mitophagy and mitochondrial energy metabolism. Conversely, overexpression of PACS2 reverses these effects to maintain right heart function (65). However, the precise molecular mechanisms underlying how PACS2 collaborates with proteins such as FUNDC1 to regulate Ca^2+^ flux, mitophagy, and cardiac function remain unclear. Böckler and Zou demonstrated that mitophagy requires Ca^2+^ flux through MAM, and artificially tethering the ER and mitochondria can rescue mitophagy defects (66, 67). Some researchers have found that decreased ATP production by inhibiting MCU-mediated Ca^2+^ influx enhances mitophagy and provides cardiac protection in heart failure (68). Thus, there is still debate regarding the impact of Ca^2+^ flux on mitophagy.

### 3.5 Other pathological cardiac hypertrophy

During pathological hypertrophy, significant changes occur in the heart's energy metabolism, accompanied by insulin resistance. _m_Ca^2+^ uptake plays a pivotal role in insulin signaling and cardiomyocyte metabolism. Insulin triggers the release and transportation of Ca^2+^ from the ER to mitochondria (69). In the norepinephrine-induced cardiomyocyte hypertrophy model, an increased distance between the ER and mitochondria hampers insulin-induced _m_Ca^2+^ uptake, Akt phosphorylation, glucose uptake, and oxygen consumption. This highlights the pivotal role of ER-mitochondrial communication in the pathogenesis of cardiac hypertrophy and related metabolic dysfunction (70). Moreover, pressure overload-induced cardiac remodeling mice exhibit elevated expression of TRPV1. TRPV1 promotes the formation of MAM, acting as a safeguard against cardiac hypertrophy. Capsaicin, a TRPV1 agonist, triggers the phosphorylation of AMP-activated protein kinase (AMPK) and upregulates MFN2 expression, facilitating MAM formation, which mitigates the cardiomyocyte size enlargement induced by adrenaline and enhances mitochondrial function. However, disruption of MAM via siMFN2 eliminates the protective effects of TRPV1 on mitochondria (71). In summary, maintaining MAM function has the potential to improve cardiomyocyte hypertrophy.

### 3.6 Heart failure

Nip3-like protein X (Nix), a protein that promotes cellular apoptosis, exhibits increased transcription levels during cardiac hypertrophy (72, 73). A recent study showed that Nix can initiate tethering of the ER to mitochondria, enhance Ca^2+^ transport, and establish “Ca^2+^ hot spots”. This mechanism leads to programmed apoptosis and necrosis in cardiomyocytes, directly contributing to the development of heart failure (26).

IP3R has three subtypes, and IP3R2 is the primary subtype in the heart (74). The interaction between FUNDC1 and IP3R2 within the MAM is crucial in maintaining mitochondrial dynamics and functionality. Wu et al. found that disrupting the FUNDC1-IP3R2 interactions within the MAM leads to decreased Ca^2+^ levels in both mitochondria and cytoplasm. This disruption ultimately results in mitochondrial dysfunction and contributes to heart failure (67). Heart-specific knockout of FUNDC1 impairs the structural integrity of MAM and reduces IP3R2 levels, causing mitochondrial elongation and mitochondrial dysfunction. Moreover, FUNDC1 regulates the expression of the Fis1 gene and controls mitochondrial fission through the Ca^2+^/cAMP response element-binding protein (CREB) pathway. Compared to healthy hearts, individuals with heart failure exhibit a more elongated morphology and reduced levels of MAM-associated proteins, including IP3R1, IP3R3, FUNDC1, phosphorylated CREB, and Fis1, resulting in a significant decrease in the proportion of adjacent ER to mitochondria. Paradoxically, these findings, in conjunction with the insights from the literature (42), highlight that the systemic and cardiac-specific knockout of FUNDC1 can yield different effects in different diseases. Further investigation into potential mechanisms is necessary, providing valuable paths for therapy.

Mitochondrial ATP-dependent Lon protease 1 (LonP1) (75) primarily resides within the mitochondrial matrix, where it acts as a crucial molecular chaperone, facilitating mitochondrial protein transport, protein folding, and unfolding processes. Additionally, LonP1 functions as a protease responsible for regulating mitochondrial protein balance, thereby playing a pivotal role in sustaining normal mitochondrial function. More recently, a study showed that LonP1 was a novel MAM tethering protein. Selectively knocking out LonP1 in mouse cardiomyocytes can disrupt the integrity of MAM, which substantially reduces interactions between mitochondria and the ER, marked by a significant decrease in IP3R3 expression levels (reduced to approximately 63% of the WT group). MAM dysfunction affects the ER unfolded protein response, although the precise mechanisms remain unclear (75). Moreover, a deficiency in LonP1 within the heart triggers metabolic reprogramming in cardiomyocytes, significantly upregulating genes associated with gluconeogenesis and amino acid metabolism, contributing to pathological heart failure. Targeting LonP1 presents a promising therapeutic approach for the treatment of heart failure.

### 3.7 Cardiotoxicity

Cardiotoxicity linked to anticancer agents is an important complication. Sorafenib (Sor), primarily used for renal cell carcinoma and hepatocellular carcinoma, stimulated the biogenesis of MAM and led to a significant increase in the essential MAM components PACS2 and FUNDC1 in cardiomyocytes. Remarkably, FUNDC1 knockdown impeded sorafenib-induced mitochondrial Ca^2+^ efflux, necrotic apoptosis, and Ca^2+^/calmodulin-dependent protein kinase II delta (CaMKIIδ) expression, highlighting the role of MAM-mediated Ca^2+^ efflux in initiating necrotic apoptosis of cardiomyocytes. Sor also reduces MFN2 protein expression in a concentration-dependent manner. MFN2 overexpression disrupts MAM components and mitochondria-ER connections, lowering activated CaMKII in cardiomyocytes, while silencing MFN2 enhances MAM components, shortens the distance, and activates CaMKII. Overall, both global and cardiac-specific MFN2 overexpression alleviate sorafenib-induced cardiac dysfunction and cardiomyocyte necrosis by inhibiting the MAM-CaMKIIδ-receptor-interacting protein kinase 3 (RIP3)/mixed-lineage kinase domain-like protein (MLKL) pathway (76). In addition, MAM is also involved in heavy metal-induced cardiotoxicity. Exposure to heavy metals such as molybdenum (Mo) and/or cadmium (Cd) can result in morphological myocardial damage, impaired oxidative function, significantly reduced _m_Ca^2+^, and an increased distance of MAM. The disrupted MAM structure is accompanied by lower levels of MAM-related genes, including IP3R, FUNDC1, MFN2 and VDAC1 (77). Combined exposure to Mo and Cd exacerbates these effects (77). Controversially, GRP75 levels rise, which can facilitate reactions in mitochondrial energy metabolism (78). Moreover, Mo and Cd can induce autophagy in sheep hearts, which may be related to MAM.

### 3.8 Sepsis-induced myocardial dysfunction

Sepsis-induced myocardial dysfunction (SIMD) is a significant complication (79, 80), with a higher mortality rate compared to patients without SIMD (70%–90% vs. 20%) (81). FUNDC1 plays a pivotal role in lipopolysaccharide (LPS)-induced myocardial dysfunction, and LPS also increases phosphorylated signal transducer and activator of transcription 3 (p-STAT3) and FUNDC1 expression in H9c2 cells. Deleting FUNDC1 interferes with MAM formation, leading to lowered intracellular Ca^2+^ and ATP levels, mitochondrial dysfunction, and decreased ROS production in LPS-injured H9c2 cells. STAT3 serves as a potential transcription factor for FUNDC1, with siSTAT3 significantly inhibiting FUNDC1 levels, while STAT3 overexpression exerts the opposite effect (82). FUNDC1 deficiency inhibits LPS-induced phosphorylation of IP3R (Ser 1,756). Stattic (a STAT3 inhibitor) suppresses the levels of the MAM-related proteins FUNDC1 and p-IP3R induced by LPS in H9c2 cells. Likewise, the interleukin-6 (IL-6)/STAT3 inhibitor bazedoxifene reduces FUNDC1 and p-IP3R levels in the hearts of LPS-treated mice. Thus, the IL-6/STAT3 signaling pathway exacerbates SIMD by promoting MAM formation dependent on FUNDC1 (82). Moreover, FUNDC1-dependent MAM formation may influence intracellular Ca^2+^ levels, in part, by impacting Cav1.2 and RyR2 levels. Nonetheless, LPS administered at a dose of 5 mg/kg significantly reduces the formation of MAM in hearts (83). These varying study outcomes result from different disease stages in sepsis (12 h vs. 18 h), emphasizing the necessity to adjust interventions based on the progression of SIMD.

### 3.9 Exercise-induced cardioprotection

Emerging evidence suggests that exercise, or exercise preconditioning (EP), potentially contributes to cardiac protection by modulating MAM proteins and functions (84). For instance, EP exerts its protective effects by regulating various molecules, including MFN2, MFN1, FUNDC1, VDAC1, GRP75, IP3R, CYPD, and others, all impacting MAM function. Among these, MFN1 and MFN2 play roles in mediating mitochondrial fusion and mitophagy, and they accumulate significantly in failing hearts. Eight weeks of exercise reduces the accumulation of MFN1 and MFN2 in failing hearts, restoring the balance of mitochondrial fission and fusion and decreasing the accumulation of mitochondrial fragments, ultimately providing cardiac protection (85). Coincidentally, six weeks of exercise improved mitochondrial dynamics by decreasing phosphorylated dynamin-related peptide 1 (Drpl)/Drp1 levels and increasing MFN2/VDAC levels in obese hearts (86). Consequently, MFN2 or MFN1 may be implicated in exercise-induced cardioprotection, and the precise regulatory mechanisms require further elucidation.

### 3.10 Cardiac aging

The prolonged accumulation of aging cardiomyocytes can detrimentally impact cardiac function, yet the associated molecular mechanisms are complicated. Recent findings suggest a connection between alterations in MAM function and cardiac aging. CDGSH iron sulfur domain 2 (Cisd2), a gene that promotes longevity, is notably abundant within MAM (87). Elevated Cisd2 levels are crucial in preserving and dynamically regulating energy metabolism. They also reduce the accumulation of myocardial lipofuscin, ultimately preserving myocardial ultrastructure and improving cardiac function related to aging. Notably, the overexpression of Cisd2 can reverse age-related cardiac functional impairments. Conversely, Cisd2 knockout leads to premature aging phenotypes, a shortened lifespan, mitochondrial dysfunction, cellular solute Ca^2+^ homeostasis disruptions, increased ROS production, and impaired autophagy (87).

## 4 Perspectives and conclusions

**Figure 3:**
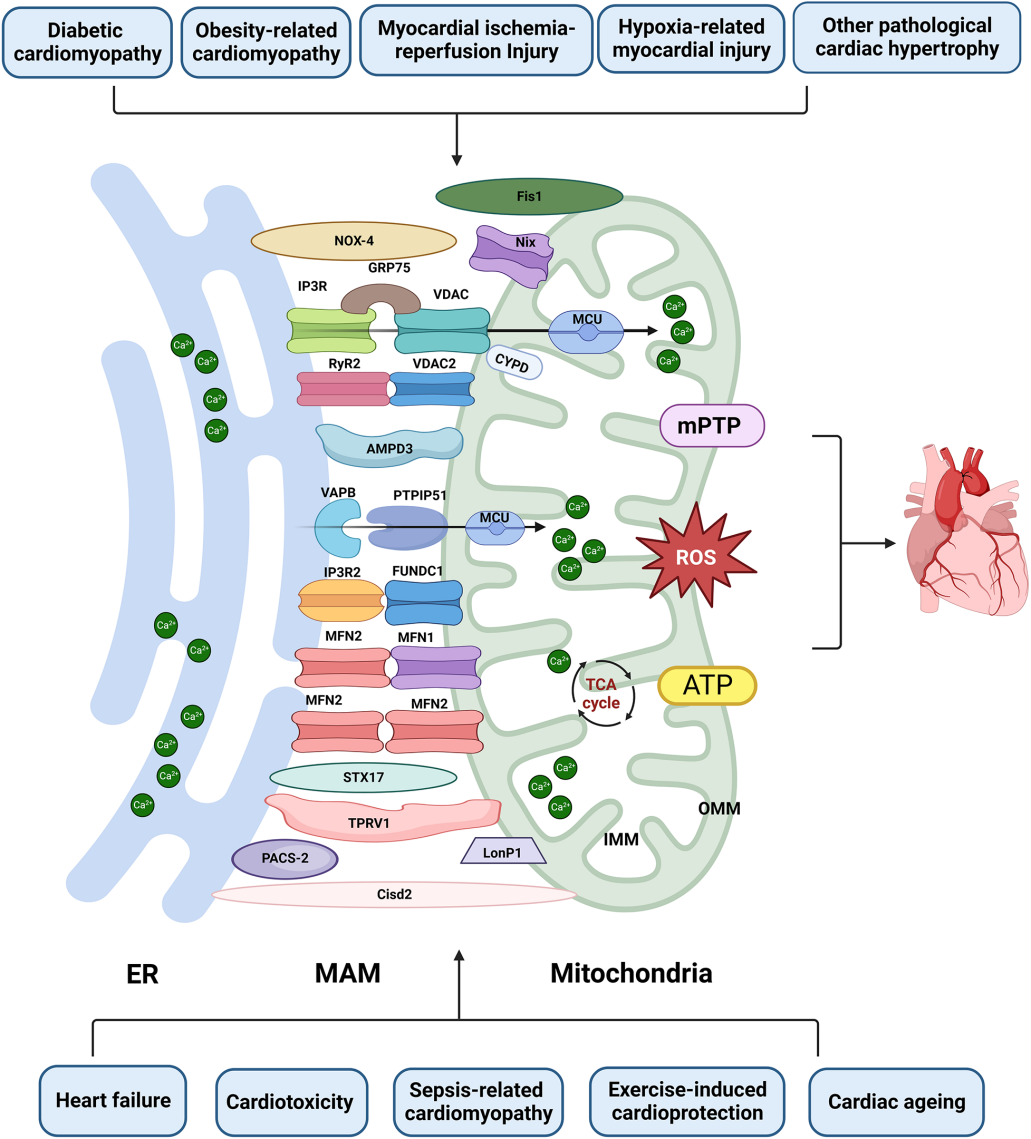
The role of MAM in cardiac disease. Proteins located on the ER surface, such as IP3R, VAPB, MFN2, and RyR2, interact with their counterparts on the OMM, including VDAC/FUNDC1, PTPIP5, MFN1/2, and VDAC2. These interactions form major tethering complexes at the MAM in cardiomyocytes. Additionally, PACS-2, NOX4, Nix, STX17, PACS-2, LonP1, TPRV1, Fis1 and Cisd1 are also involved in the structure and functions of MAM. Dysfunction of MAM can lead to Ca^2+^ overload, abnormal ROS and ATP production, subsequently inducing mPTP opening and apoptosis. This dysfunction is implicated in various types of heart injuries. Created with BioRender.com.

The interaction between mitochondria and the ER has garnered significant attention in the past decade. However, our current understanding of the composition and function of MAM remains incomplete and subject to controversy. Paillard et al. reported that acute disruption of MAM (achieved by inhibiting GRP75) could protect the heart from I/R injury by preventing _m_Ca^2+^ overload, mPTP opening, and cell apoptosis (52). The same team recently showed that MAM disruption, leading to reduced Ca^2+^ transfer, could induce early mitochondrial dysfunction and contribute to DbCM (33). In addition, Papanicolaou discovered that cardiomyocytes become more resistant to cell death when MFN2 is knocked out, as they exhibit increased tolerance to Ca^2+^-induced mPTP opening (88). Nevertheless, Filadi R reported that MFN2 gene knockout or silencing displayed increased tethering between the ER and mitochondria, enhanced Ca^2+^ transfer, and increased sensitivity to apoptotic stimuli associated with _m_Ca^2+^ overload toxicity (89). MFN2 disruption additionally worsened ER/mitochondrial metabolic feedback, leaving ventricular cardiomyocytes from MFN2 knockout mice incapable of inducing ATP production through IP3-mediated ER Ca^2+^ release. Furthermore, MFN2-mediated ER-mitochondrial communication varied depending on mitochondrial position and receptor types, which may help explain the controversies in this field (90). In addition, FUNDC1 deficiency leads to heart failure due to reduced formation of MAM and decreased _m_Ca^2+^ levels (67). In contrast, FUNDC1 KO mitigates cardiac function abnormalities in Akita and streptozotocin-induced diabetes, primarily due to reduced MAM formation and lower _m_Ca^2+^ levels (31). Nevertheless, the absence of FUNDC1 exacerbates mitochondrial dysfunction and Ca^2+^ overload in obesity-related cardiomyopathy (42). The MAM regulation on mitochondrial dynamics is regulated by numerous functional regulatory pathways, forming a complex spatial and temporal regulatory network that is currently not fully understood. The combinations of different molecules exhibit distinctly different MAM regulatory effects, which may be influenced by specific disease models, injury time, MAM assessment methods, and the involved MAM molecules. Hence, comprehending the importance of diverse pathological stimuli, mitochondrial localization, MAM biogenesis, and other factors in inducing MAM changes is crucial for the precise regulation of MAM adaptive homeostasis (Figure 3 and Table 1).

**Table 1:** The function and expression of MAM components in different cardiac diseases.

Cardiac diseases	Protein involved	Function	Model	Level	Impact
DbCM	FUNDC1/IP3R2 (31)	Ion transport	Human diabetes patients; streptozotocin-treated cardiac-specific FUNDC1 knockout Akita mice; rat H9c2 cardiomyoblasts	↑	Upregulation of FUNDC1, IP3R2 increased _m_Ca^2+^ concentration, which leaded to mitochondrial fragmentation, mPTP opening, and cardiomyocyte apoptosis.
	IP3R/GRP75/VDAC (33)	Ion transport	HFHSD induced diabetes mice	↓	There was a decline in Ca^2+^ transfer and mitochondrial bioenergetics, which resulting in reduced cardiomyocyte contractility.
	MFN2 (35)	Tethering	Primary atrial myocytes from Sprague-Dawley rats	↑	The reduced distance of MAM resulted in Ca^2+^ overload, which is related to mitochondrial dysfunction and ERS-induced apoptosis.
	AMPD3 (36)	Tethering	OLETF; H9c2 cells	↑	The MAM formation, _m_Ca^2+^ overload, mitochondrial dysfunction are promoted, and ATP production is impaired.
Obesity-related cardiomyopathy	MFN2 (41)	Tethering	Long-term west diet feeding mice	↓	ER-mitochondria communication was disrupted.
	FUNDC1 (42)	Mitophagy	High fat diet mice	↓	Loss of FUNDC1 diminished degradation of IP3R3, accentuated mitochondrial anomalies, cell death, and Ca^2+^ overload.
	STX17 (45)	Tethering	High fat diet mice	↑	The STX17 promoted _m_Ca^2+^ overload, which exacerbated cardiac oxidative stress, mitochondrial damage, and functional impairments.
I/R	CYPD (52)	Regulatory	H9c2 cells; adult mice cardiomyocytes	↑	The CYPD regulated the _m_Ca^2+^ load and mPTP opening via interacting with the VDAC1/GRP75/IP3R1 complex.
	NOX4 (55)	Tethering	Primary cardiomyocytes of rats; rat H9c2 cardiomyoblasts; Nox4 knockout mice	↑	NOX4 enhanced Akt-dependent phosphorylation of IP3R1, consequently inhibiting Ca^2+^ flux and mPTP-dependent necrosis.
	PTPIP51/VAPB (59)	Tethering	C57BL/6 mice; primary cardiomyocytes of rats	↑	PTPIP51/VAPB enhanced MAM-mediated _m_Ca^2+^ uptake and induce apoptosis.
	TRPV1 (60)	Ion transport	Rat H9c2 cells	–	Activation of TRPV1 mobilized Ca^2+^ from ER to mitochondria and remodeled MAM.
Hypoxia-related myocardial injury	VDAC/IP3R1 (64)	Ion transport	Atrial tissue of SDB patients; Swiss/SV129 mice; H9c2 cardiomyoblasts	↓	The interaction of VDAC/IP3R1 was mediated by HIF-1 and contributed to disturbances in Ca^2+^ homeostasis and myocardial cell death.
	PACS2 (65)	Tethering	C57BL/6J mice	↓	The downregulation of PACS2 disrupted MAM formation and Ca^2+^ flux, inhibited mitophagy and mitochondrial energy metabolism.
Other pathological cardiac hypertrophy	TRPV1 (71)	Ion transport	C57BL/6 J TAC mice; phenylephrine treated neonatal rat cardiomyocytes	↑	The TRPV1 upregulated MFN2 expression, promoted MAM formation and mitochondrial function, and mitigates the cardiomyocyte size enlargement.
Heart failure	Nix (72, 73)	Tethering	Cardiac-specific G*α*q transgenic mouse; phenylephrine treated neonatal rat cardiomyocytes	↑	The Nix enhanced Ca^2+^ transport and promoted cellular apoptosis.
	FUNDC1/IP3R2 (67)	Ion transport	Heart failure patients; cardiomyocyte-specific FUNDC1 knockout C57BL/6J mice; mice neonatal cardiomyocytes	↓	The downregulation of FUNDC1/IP3R2 decreased Ca^2+^ levels both in mitochondria and cytoplasm, causing mitochondrial elongation and dysfunction.
	LonP1 (75)	Tethering	Heart of LonP1 conditional knockout mice; H9c2 rat embryonic cardiomyocyte	–	Knocking out LonP1 disrupted the integrity of MAM, induced metabolic reprogramming and mitochondrial fragmentation, and decreased IP3R3 expression.
Cardiotoxicity	FUNDC1 (76)	Ion transport	Sorafenib induced C57BL/6 mice; primary mouse cardiomyocytes	↑	Knock down FUNDC1 impeded sorafenib-induced mitochondrial Ca^2+^ efflux, necrotic apoptosis, and CaMKIIδ expression.
	MFN2 (76)	Tethering	Sorafenib induced C57BL/6 mice; primary mouse cardiomyocytes	↓	Silencing MFN2 enhanced MAM components, shortened the distance, and activated CaMKIIδ.
	IP3R/FUNDC1/VDAC1/MFN2 (77)	Ion transport/tethering	Molybdenum and cadmium induced sheep	↓	The molybdenum and/or cadmium increased distance of MAM and reduced IP3R/FUNDC1/VDAC1/MFN2 expressions and _m_Ca^2+^.
Sepsis-induced myocardial dysfunction	FUNDC1 (82)	Ion transport	LPS treated C57BL/6J mice; H9c2 cells and AC16 cells	↑	Deleting FUNDC1 interfered with MAM formation, leading to lowered intracellular Ca^2+^, ROS and ATP levels, mitochondrial dysfunction, and inhibited LPS-induced phosphorylation of IP3R.
Exercise-induced cardioprotection	MFN2/MFN1 (85)	Tethering	Myocardial infarction-induced heart failure Wistar rats	↑	The exercise reduced the accumulation of MFN1 and MFN2 in failing hearts, restoring the balance of mitochondrial fission and fusion and decreasing the accumulation of mitochondrial fragments.
Cardiac aging	Cisd2 (87)	Tethering	Cisd2 transgenic and wild type mice	↓	The knockout of Cisd2 leaded to premature aging phenotypes, a shortened lifespan, mitochondrial dysfunction, cellular solute Ca^2+^ homeostasis disruptions, increased ROS production, and impaired autophagy.

↑, upregulation;↓, downregulation; -, not applicable; AMPD3, adenosine monophosphate deaminase 3; Akt, protein kinase B; ATP, adenosine triphosphate; CaMKIIδ, Ca^2+^/calmodulin-dependent protein kinase II delta; Cisd2, CDGSH iron sulfur domain 2; CYPD, cyclophilin D; DbCM, diabetic cardiomyopathy; ERS, endoplasmic reticulum stress; FUNDC1, FUN14 domain containing 1; GRP75, glucose-regulated protein 75; HFHSD, high-fat, high-sucrose diet; HIF-1, hypoxia-inducible factor 1; IP3R, inositol 1,4,5-trisphosphate receptor; LPS, lipopolysaccharide; LonP1, lon protease 1; MFN2, mitofusin 2; mPTP, mitochondrial permeability transition pores; Nix, nip3-like protein X; NOX4, NADPH oxidase 4; OLETF, otsuka long-evans tokushima fatty; PACS-2, phosphofurin acidic cluster sorting protein 2; PTPIP51, protein tyrosine phosphatase interacting protein 51; ROS, reactive oxygen species; SDB, sleep-disordered breathing; STX17, syntaxin 17; TRPV1, transient receptor potential cation channel subfamily V member 1; VAPB, vesicle-associated membrane protein-associated protein B; VDAC, voltage-dependent anion channel.

Apart from the proteins, the physical connection between the ER and mitochondria MAM, also known as the mitochondria-endoplasmic reticulum contact (MERC) tether, holds promise as a therapeutic target (91). Prior research has demonstrated that compounds such as arachidonic acid or melatonin, which intervene in the function of MERC, can ameliorate DbCM (32) and myocardial damage in type 3 cardiorenal syndrome (92). Since endogenous mitochondrial-ER tether proteins serve multiple functions, genetically targeting them is unsuitable for selectively investigating specific tether functions. A previous study showed that an artificial ER-mitochondria connector formed a single-protein bridge between the OMM and ER, resulting in a more robust and expansive organelle association (91). The heart-specific engineered tether in mice can enhance MERC, leading to long-term adaptations that sustain excitation-energy coupling. Concurrently, enhanced interactions lessened the susceptibility of female mice to β-adrenergic stress and alleviated myocyte death and myocardial dysfunction induced by _m_Ca^2+^ overload during I/R injury (93). Therefore, the MERC tether not only facilitates the investigation of specific roles of mitochondrial-ER interactions in the heart but also offers the potential to examine their overall impact on tissues. Further exploration of the long-lasting effects of continuous enhancement of cardiac mitochondrial ER tethering opens up possibilities for utilizing structural preconditioning as a genetic therapeutic strategy.

In general, ER and mitochondrial networks undergo highly dynamic changes, which are capable of altering their shapes and/or distribution in response to various stimuli. The integrity of the MAM forms the foundation for effective signal transmission and uniquely regulates cardiomyocyte mitochondrial homeostasis, apoptosis, and autophagy (3). Therefore, analyzing MAM length, the distance between the two organelles, the number of contact points, communication types, and persistence at different stages of diseases can help elucidate the potential therapeutic value of targeting MAM in preventing cardiac disease. Despite recent achievements, there is still a long way to go.
